# Pathogens that Cause Illness Clinically Indistinguishable from Lassa Fever, Nigeria, 2018

**DOI:** 10.3201/eid2805.211153

**Published:** 2022-05

**Authors:** Jonathan W. Ashcroft, Adebola Olayinka, Nnaemeka Ndodo, Kuiama Lewandowski, Martin D. Curran, Chioma Dan Nwafor, Kingsley Madubuike, Helen Bagnall, Abdulmajid S. Musa, Adama Ahmad, Afolabi Akinpelu, Adesola Adeleye, Chukwuji Martin, Adejoke Akano, Michael Onoja, Akanimo Iniobong, Winifred Ukponu, Chinedu Arinze, Else Ilori, Nwando Mba, Benedict Gannon, Anthony Ahumibe, Chikwe Ihekweazu

**Affiliations:** Public Health England, London, UK (J.W. Ashcroft, H. Bagnall, B. Gannon);; London School of Hygiene and Tropical Medicine, London (J.W. Ashcroft, H. Bagnall, B. Gannon);; Nigeria Centre for Disease Control, Abuja, Nigeria (A. Olayinka, N. Ndodo, C. Dan Nwafor, K. Madubuike, A.S. Musa, A. Ahmad, A. Akinpelu, A. Adeleye, C. Martin, A. Akano, M. Onoja, A. Iniobong, W. Ukponu, C. Arinze, E. Ilori, N. Mba, A. Ahumibe, C. Ihekweazu);; Public Health England, Porton Down, UK (K. Lewandowski);; Clinical Microbiology and Public Health Laboratory, Public Health England, Cambridge, UK (M.D. Curran)

**Keywords:** Lassa fever, Lassa virus, sequencing, TaqMan Array Card, differential diagnosis, West Africa, Nigeria, viruses

## Abstract

During the 2018 Lassa fever outbreak in Nigeria, samples from patients with suspected Lassa fever but negative Lassa virus PCR results were processed through custom gene expression array cards and metagenomic sequencing. Results demonstrated no single etiology, but bacterial and viral pathogens (including mixed co-infections) were detected.

Timely and accurate laboratory differentiation of infectious agents responsible for acute febrile illness represents a major challenge for West Africa. The etiology of systemic febrile illness is particularly poorly described; numerous region-endemic diseases lead to similar initial clinical signs and symptoms (*1*).

In 2018, Nigeria experienced its largest recorded outbreak of Lassa fever; during January 11–December 31, 2018, a total of 3,498 suspected cases were reported. Of these, 633 were confirmed positive, 20 probable, 2,853 negative, and 8 undetermined (*2*). A high number of patients met the case definition for Lassa fever yet ultimately tested negative for the virus and no causative pathogen was identified. To determine the causes of the patients’ illnesses, we analyzed gene expression and conducted metagenomic analysis. Ethics approval was obtained from the London School of Hygiene and Tropical Medicine Ethical Review Board (reference no. 16263) and National Health Research Ethics Committee of Nigeria (reference no. NHREC/01/01/2007–19/03/2019).

## The Study

During January–December 2017, state health departments across Nigeria collected a total of 160 samples according to Nigeria’s National Lassa Fever Outbreak Guidance for patients who met the case definition for Lassa fever (V. Navapurkar et al., unpub. data,  https://www.medrxiv.org/content/10.1101/2020.06.02.20118489v3.full.pdf) (Appendix). For sample selection, we used a convenience-based approach. Inclusion criteria were sample collection in 2018, Lassa-negative quantitative reverse transcription PCR (RT-PCR) results, malaria-negative test results (CareStart Malaria RDT; AccessBio, https://accessbio.net), sufficient sample remaining for subsequent testing, and available basic patient demographic information.

To address the differential diagnoses, we opted to use a TaqMan Array Card (Applied Biosystems, https://www.thermofisher.com) with prespotted singleplex real time PCRs (1 sample can be simultaneously screened for 50 pathogens) (Appendix Figure 2). The assay and data analysis were conducted as previously described (*3*,*4*; S. Minot et al., unpub. data, https://www.biorxiv.org/content/biorxiv/early/2015/09/28/027607.full.pdf). We visually inspected the amplification curve of each reaction and classified findings as positive (pathogen target detected) or negative (pathogen target not detected). We used no multicomponent or raw data plots for classification. Public Health England (Cambridge) independently reviewed the final results and found no deviations in reported interpretations. We randomly selected 12 of the samples that had been run on the TaqMan Array Cards (TACs) and subjected them to MinION sequencing by previously described methods (*5*,*6*). Of these 12, we found 0 positive TAC hits for 3 samples and 1–8 hits for the remaining 9 samples.

We examined samples collected from 21 of 37 states within Nigeria, most from Plateau (20.00%), Bauchi (15.60%), Nasarawa (11.25%), Federal Capital Territory (10.00%), Taraba (8.75%), and Kogi (6.88%). Of the 160 samples, ≈58% were from male patients; combined population ages ranged from 2 months to 70 years (median age for male and female patients was 25 years) (Appendix Table 1).

**Figure 1:**
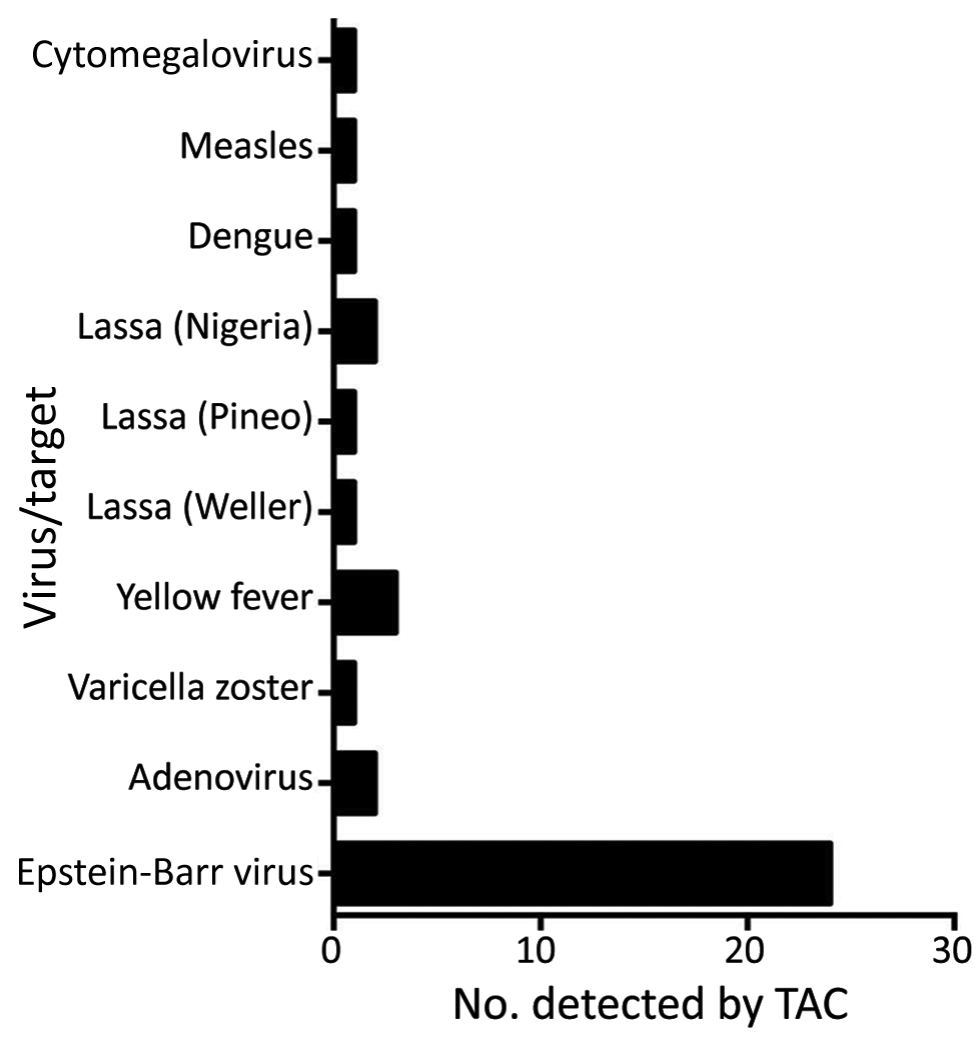
Number of samples from patients who met the case definition for Lassa fever that were positive for specific viral pathogens, among 160 samples tested, Nigeria, 2018. CMV, cytomegalovirus; TAC, TaqMan Array Cards (Applied Biosystems, https://www.thermofisher.com).

**Figure 2:**
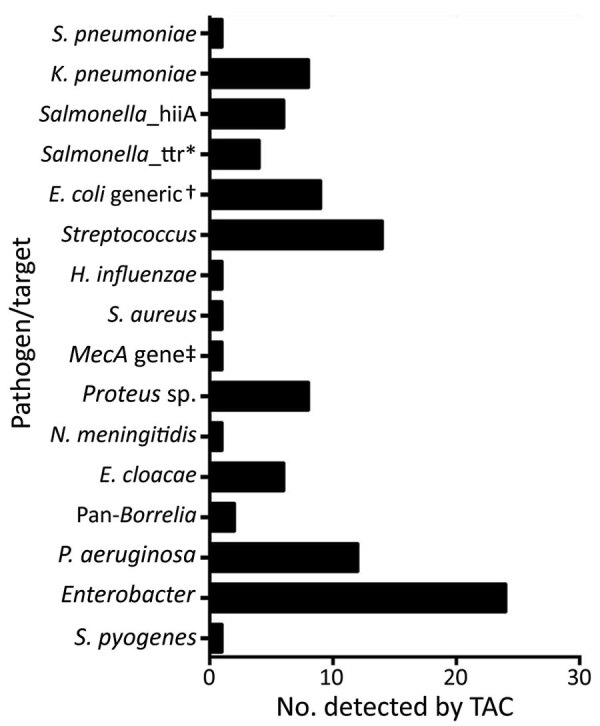
Number of samples from patients who met the case definition for Lassa fever that were positive for specific bacterial pathogens, among 160 samples tested, Nigeria, 2018. *All *Salmonella*_ttr–positive samples also registered as *Salmonella*_hilA–gene positive. †Includes one EAggEC. ‡Sample also positive for *Streptococcus*, *Proteus* spp., and *Pseudomonas aeruginosa*. *E. cloacae*, *Enterobacter* *cloacae*; EAggEC, enteroaggregative *E. coli*; *E. coli*, *Escherichia coli*; *H*., *Haemophilus*; *K*., *Klebsiella*; *N*., *Neisseria*; *P*., *Pseudomonas*; *S*., *Streptococcus*; TAC, TaqMan Array Cards (Applied Biosystems, https://www.thermofisher.com); ttr, tetrathionate.

Of the 160 samples tested, TAC detected >1 positive bacterial or viral hit for 84 (52.5%) samples. TAC runs recorded positive hits for 8 viruses and 15 types of bacteria (Figures 1, 2; Appendix Figure 3). Virus results were positive for Lassa virus, yellow fever virus, measles virus, cytomegalovirus, adenovirus, Epstein-Barr virus, dengue virus, and varicella zoster virus. The most prevalent species of bacteria among the 15 identified were *Streptococcus* spp., *Salmonella* spp., *Enterobacteriaceae* spp., *Pseudomonas aeruginosa*, *Escherichia coli*, and *Klebsiella pneumoniae*. Cycle threshold (Ct) ranges for the positive hits ranged from a low of 16.2 (*Neisseria meningitidis*) to a high of 43.8 (*Proteus* spp.); Ct for most samples was in the 25–35 range (Appendix Figure 4).

Of the 84 samples positive by TAC, 34 registered >1 target, including mixed bacterial and viral infections (Appendix Table 2, Figure 3). Of these 84, most (95.23%) contained 1–4 detectable pathogens; the remaining samples (4.8%) contained 5–7 detectable pathogens. The pathogenic constellations of patients with higher levels of co-infection (e.g., Epstein-Barr virus, *K. pneumoniae*, and *Enterobacter cloacae*) are in line with those expected to be observed in immunocompromised persons (*7*). Although confident with the results, we cannot completely rule out the possibility of sample contamination; however, we took steps to minimize contamination (e.g., we prepared fresh RNA extractions in dedicated cabinets and used sample tracking forms).

For confirmatory sequencing using the MinION sequencing platform, we randomly selected a subset of 9 TAC-positive and 3 TAC-negative samples. Sequencing was performed in Nigeria (National Reference Laboratory, Abuja, Nigeria) and in the United Kingdom (Public Health England, Porton Down, UK). The MS2 control spike, used to demonstrate reverse transcription and sequencing efficiency, was satisfactory in all samples.

With respect to viral pathogens, the sequencing data confirmed the results registered by the TACs where available (Table 1). The only differences observed were for 2 samples: 1 weakly positive (Ct >40) for dengue but not detected via sequencing and 1 negative by TAC but proven positive for pegivirus C (a pathogen not represented on the TAC).

**Table 1:** Array and MinION sequencing results for a subset of samples from patients who met the case definition for Lassa fever that were positive for virus, Nigeria, 2018

Sample	Kraken hits	TAC virus hits	Mapping hits	Mapped reads, no. (%)
307	None	None	NA	NA
165	Human mastadenovirus B	Adenovirus	Adenovirus 2	246 (0.07)
349	Yellow fever virus	Yellow fever virus	Yellow fever virus	66 (0.02)
370	None	None	NA	NA
184	None	None	NA	NA
320	None	Epstein-Barr virus	Epstein-Barr virus	22 (0.01)
344	None	None	NA	NA
279	Yellow fever	Yellow fever	Yellow fever virus	72 (0)
157	Pegivirus C (hepatitis G)	None	Pegivirus C (hepatitis G)	116 (0.01)
147	None	Dengue 2 virus	Dengue 2 virus	0
322	None	None	NA	NA
70	Lassa virus	Lassa virus	Lassa virus	16309 (5.05)-L, 8265 (2.575)-S
201	None	None	NA	NA

*MinION described in (*5**,**6*). NA, not applicable; TAC, TaqMan Array Cards (Applied Biosystems, https://www.thermofisher.com).

Of note are the yellow fever virus–positive and Lassa virus–positive results. The yellow fever virus–positive samples were from Kaduna and Kogi states; patients first displayed signs/symptoms in late July, late August, and early November 2018, the year when the Nigeria Centre for Disease Control reported a large and widespread outbreak of yellow fever in Nigeria, which affected many states. Centre data indicate that, at the time of collection of the 3 yellow fever samples that were positive by TAC with or without sequencing, those states had neither suspected nor confirmed cases of yellow fever (*8*). As such, our results confirm the presence of yellow fever virus when presence of the disease was only suspected.

For the 3 Lassa virus–positive samples, 1 had been misclassified as negative because of an initial laboratory error (e.g., undetected run fail). Of the other 2 samples, 1 was originally recorded as negative by RT-PCR, but a rerun confirmed the presence of Lassa virus (Altona, Ct = 38.51); the other registered as positive for Lassa virus (Nigeria, Pinneo strain, clade 1) but did not register a positive result on RT-PCR (Altona), possibly because of diagnostic primer sets not possessing sufficient homology.

With respect to samples that contained TAC-positive bacterial targets, because sample extracts had been prepared to favor detection of viral pathogens, we could not complete full analysis of potential bacterial pathogens. We compared potential bacterial pathogens indicated by TAC with the Kraken (https://github.com) taxonomic analysis. Read numbers were reported at the genus level (Table 2). Analysis does not rule out the presence of these pathogens; however, data are insufficient for determining presence with certainty.

**Table 2:** Array and MinION sequencing results for a subset of samples from patients who met the case definition for Lassa fever that were positive for bacteria, Nigeria, 2018*

Sample	TAC hits	Seq hit 1 (no. mapped reads)	Seq hit 2 (no. mapped reads)	Seq hit 3 (no. mapped reads)	Seq hit 4 (no. mapped reads)	Seq hit 5 (no. mapped reads)
307	*Klebsiella pneumoniae*	*Salmonella*_hilA (108)	*Salmonella*_ttr (55)	*Enterobacter* (55)	*Enterobacter cloacae* (55)	*Enterobacter* (56)
165	Pan-*Borrelia*	Spirochetes (27)	None	None	None	None
349	*Escherichia coli* generic	*E. coli* (58)	None	None	None	None
344	*Streptococcus*	Mec_A (591)	*Staphylococcus* (657)	*Pseudomonas aeruginosa* (3)	*Streptococcus. pyogenes* (591)	*Streptococcus* (591)
279	*Streptococcus*	*Streptococcus* (8,837)	None	None	None	None

*Only samples with hits are shown. Seq hits at the genus level (number of reads). MinION described in references *5**,**6*. TAC, TaqMan Array Cards (Applied Biosystems, https://www.thermofisher.com). Seq hit, sequencing hit.

## Conclusions

When examining samples from patients who met the case definition for Lassa fever but tested negative for the Lassa virus by quantitative RT-PCR, we found that processing the samples through custom TaqMan Array Cards revealed that there was no single cause of the patients’ signs/symptoms. Instead, results were far more complex, detecting a variety of bacterial and viral pathogens (including mixed co-infections). For the random TAC-positive and TAC-negative samples that underwent metagenomic sequencing, results corroborated the TAC viral results well and supported the bacterial results. It is likely that a proportion of the TAC-negative samples (47.5%) were from patients whose illness did indeed have an infectious etiology but did not register on the TAC because the pathogen for the molecular target was not represented, and a proportion might not have had an infectious origin. The pathogens identified in this study could be added to the differential diagnosis for patients with Lassa fever signs/symptoms but negative Lassa virus/malaria test results during outbreaks in West Africa.

Appendix Supplemental methods and results for study of pathogens that cause illness clinically indistinguishable from Lassa fever, Nigeria, 2018.
