# Supplementary material for: Pathogens that Cause Illness Clinically Indistinguishable from Lassa Fever, Nigeria, 2018
Source: Emerg Infect Dis. 2022 May;28(5):994–7. doi: 10.3201/eid2805.211153 (PMC9045454; doi:10.3201/eid2805.211153)
Supplement: Appendix — Supplemental methods and results for study of pathogens that cause illness clinically indistinguishable from Lassa fever, Nigeria, 2018. [file 21-1153-Techapp-s1.pdf]

Article DOI: https://doi.org/10.3201/eid2805.211153

Pathogens that Cause Illness Clinically Indistinguishable from Lassa Fever, Nigeria, 2018

Appendix

NCDC Guidelines for Lassa Fever Diagnostic Specimens

Strict adherence to infection prevention and control measures is recommended throughout the specimen collection process, including waste disposal and disinfection. The following specimens are recommended for collection from live patients;

- Whole blood in EDTA
  - ➢ 5ml from adults
  - ➢ 2-3mls from children
  - ➢ 1.5ml from neonates

Depending on clinical parameters, others such as CSF, breastmilk, semen etc may be used

- The following information should be captured for every specimen collected from a patient; Name of Patient, Patient ID (Hospital number, lab number or Epid no), date of collection and State.

Timing of specimen collection

- Specimens for molecular detection should be taken when a patient exhibits symptoms that meet the case definition of Lassa Fever virus infection.

- If specimens are collected less than 3 days after onset of symptoms, additional specimens will be needed if the test result on the first specimen is negative. The second specimen should be collected at least 48 hours after the first specimen.

References

1. Wood DE, Lu J, Langmead B. Improved metagenomic analysis with Kraken 2. Genome Biol. 2019;20:257. PubMed https://doi.org/10.1186/s13059-019-1891-0
2. Li H, Durbin R. Fast and accurate long-read alignment with Burrows-Wheeler transform. Bioinformatics. 2010;26:589–95. PubMed https://doi.org/10.1093/bioinformatics/btp698
3. Endimiani A, Luzzaro F, Brigante G, Perilli M, Lombardi G, Amicosante G, et al. *Proteus mirabilis* bloodstream infections: risk factors and treatment outcome related to the expression of extended-spectrum beta-lactamases. Antimicrob Agents Chemother. 2005;49:2598–605. PubMed https://doi.org/10.1128/AAC.49.7.2598-2605.2005

Appendix Table 1. Demographics of Lassa-Negative patients whose samples are included in this study.

Group	No of samples	Age range	Median age, yrs	SD
Male	93	2 month –70 yrs	23.20	16.30
Female	67	1yr–65 yrs	25.73	16.40
Overall	160	2 months–70 yrs	25.53	16.28

Appendix Table 2. Distribution of TaqMan Array Card (TAC) positive results by the number of pathogens detected in each patient sample. *Total number of patient samples tested = 160.*

Pathogen/Sample	Number of Samples
0	76
1	50
2	18
3	11
4	1
5	3
6	0
7	1
Total	160

A suspected case describes any individual presenting with one or more of the following:	
-Malaise<br>-Fever<br>-Headache<br>-Sore throat<br>-Cough<br>-Nausea	-Vomiting<br>-Diarrhoea<br>-Myalgia<br>-Chest pain<br>-Hearing loss
And either:	
a) History of contact with excreta or urine of rodents – or –	b) History of contact with a probable or confirmed Lassa fever case within a period of 21 days of onset of symptoms OR inexplicable bleeding.

Appendix Figure 1. Nigerian National Laboratory Testing Algorithm & Case Definition for Lassa Fever Virus.

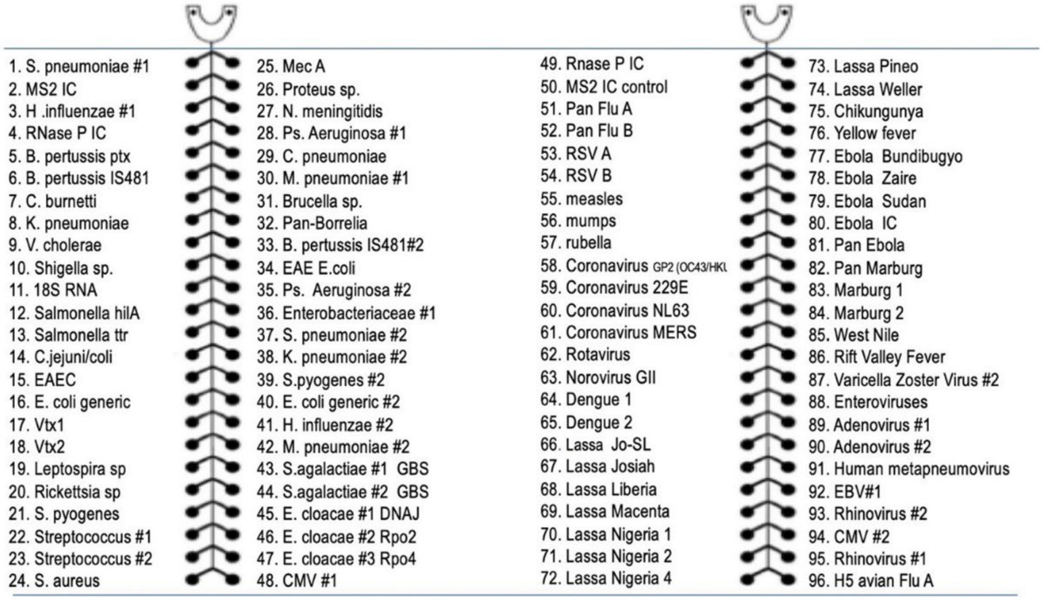

Appendix Figure 2. TaqMan Array Card Layout (West Africa Panel). This system is based on singleplex, reverse transcription real-time PCR (RT-PCR). The 384-well microfluidic card is pre-spotted with PCR reactions allowing a single sample to be simultaneously screened for up to 50 pathogens. Variants of these cards are already commercialised for multiple gene expression and micro-RNA expression analyses, have been extensively used in cancer research and have been used previously for infectious disease investigations (S. Minot et al., unpub. data, https://www.biorxiv.org/content/biorxiv/early/2015/09/28/027607.full.pdf) (*1*,*2*).

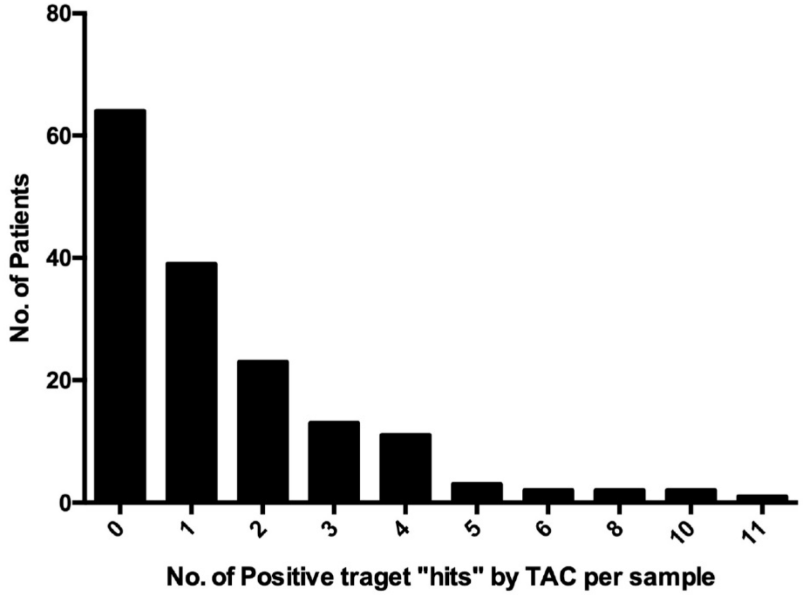

Appendix Figure 3. Distribution of TaqMan Array Card (TAC) positive results by the number of positive "hits" detected in each patient sample. *Total number of patient samples tested = 160*.

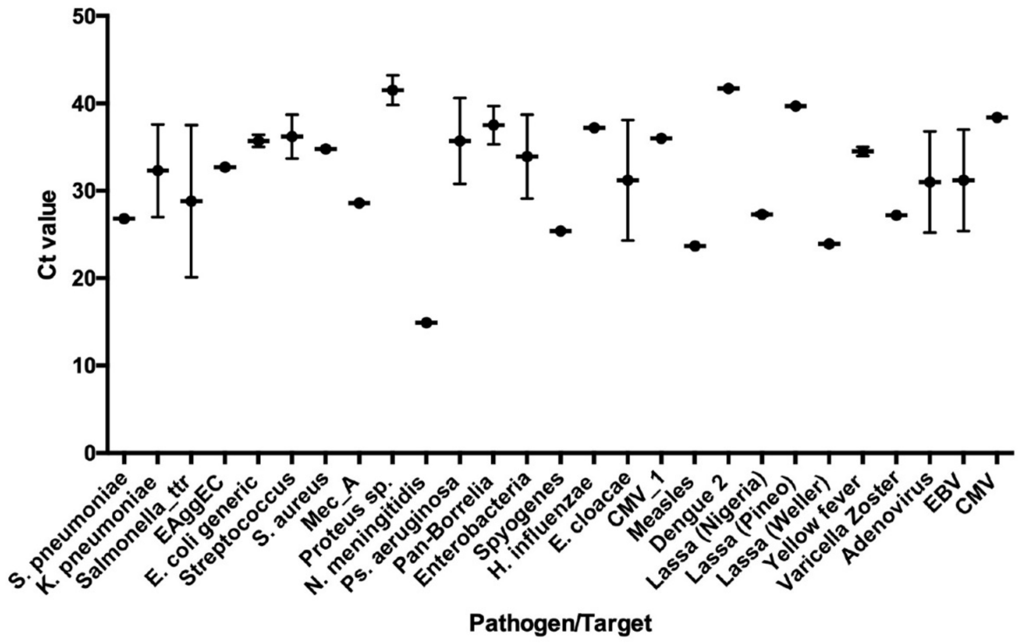

Appendix Figure 4. Ct Values from samples run on the TaqMan Array Card. Average Ct values (inc. SD) presented by pathogen/target detected in each patient sample by TaqMan Array Card (TAC). The majority of samples registering a positive "hit" fell between Ct 25-35 and ranged from a low of 16.2 (*N. meningitidis*) to a high of 43.8 (*Proteus spp.*). Although *Proteus spp* registered a high Ct, this is not considered to be an aberrant result. Bloodstream infection (BSI) due to *Proteus mirabilis* strains is a relatively uncommon clinical entity, and its significance has received little attention to-date. Patients with detectable *Proteus mirabilis* BSIs present with sepsis, severe sepsis or septic shock all of which have some clinical manifestation common to Lassa fever (*3*).
